# Effect of Microplastic Particles on the Rheological Properties of Human Saliva and Mucus

**DOI:** 10.3390/ijerph20227037

**Published:** 2023-11-08

**Authors:** Rafał Przekop, Urszula Michalczuk, Agata Penconek, Arkadiusz Moskal

**Affiliations:** Faculty of Chemical and Process Engineering, Warsaw University of Technology, 00-645 Warsaw, Poland; urszula.michalczuk.dokt@pw.edu.pl (U.M.); agata.penconek@pw.edu.pl (A.P.); arkadiusz.moskal@pw.edu.pl (A.M.)

**Keywords:** microplastics, rheology, saliva, mucus

## Abstract

Pollution by plastic microparticles is rising rapidly. One avenue of human exposure to nanoparticles is through inhalation. The main source of microplastics in indoor environments, leading to unintended inhalation, is synthetic fabric used in clothing. Other sources include curtains, carpets, furniture, wall paints, and floor finishes. Occupational exposure is particularly significant in waste management and recycling operations, during exposure to high heat, during high-energy treatment of polymer composites, and during 3D printing. In outdoor environments, exposure can happen through breathing in contaminated aerosols from ocean waves or airborne particles from dried wastewater treatments. Airborne particles affect human health in various ways, including via direct interactions with the epithelium and its mucus layer after deposition in the mouth and respiratory system. Exposure due to the ingestion of microplastics present in various environmental compartments may occur either directly or indirectly via the food chain or drinking water. This study aimed to determine the effects of plastic microparticles on the rheology of mucus and saliva, and, thus, their functioning. The experiments used artificial mucus, saliva, and plastic nanoparticles (namely, PS—polystyrene and PE—polyethylene). The rheological properties of saliva and mucus were determined via the use of an oscillatory rheometer at various temperatures (namely, 36.6 °C and 40 °C, which correspond to healthy and ill humans). The results were compared with those obtained for pure saliva and mucus. An increase in apparent viscosity was observed for saliva, which is behavior typical of for solid particle suspensions in liquids. In contrast, for mucus, the effect was the opposite. The influence of the presence of the particles on the parameters of the constitutive viscosity equations was studied. Plastic micro- and nanoparticles in the saliva and mucus may interfere with their physiological functions.

## 1. Introduction

The global emission and production of plastic materials are significantly growing, leading to accumulation in the natural environment. Consequently, plastic particles are present in food [1], drinking water [2,3], and human lungs [4]. The knowledge regarding the side effects caused by plastics present in the human body still needs to be improved. In this paper, we aim to investigate the impact of the presence of microplastics on the rheological properties of artificial saliva and mucus.

Micro- and nanoplastics can be divided into primary and secondary categories. Microplastics (MPs) are considered to be primary when they are directly emitted as such into the environment, and secondary when they are degradation products from larger plastic objects (macroplastics) already in the environment. For example, macroplastics can break down due to UV light, thermal changes, and oxidative weathering, resulting in secondary microplastics [5]. Every plastic product can end up in the environment and is, therefore, a potential source of microplastics.

Liao et al. [6] studied the indoor and outdoor concentrations of microplastics in urban and rural areas of a coastal city in eastern China. The microplastic concentrations (mean value ± standard deviation) in indoor air (1583 ± 1180 particles/m^3^) were an order of magnitude higher than in outdoor air (189 ± 85 particles/m^3^). Airborne microplastic concentrations in urban areas (224 ± 70 particles/m^3^) were higher than in rural areas (101 ± 47 particles/m^3^). The study estimated that the maximum annual outdoor exposure of airborne microplastics could reach 1 million/year, while indoor exposure may be even higher due to the higher indoor concentrations of airborne microplastics. Studies on plastic microparticle concentrations in a marine environment (the Baltic Sea) have shown a concentration of 161 ± 75 particles/m^3^ in the harbor of Gdańsk, a higher value compared to the open Baltic Sea and the island of Gotland (24 ± 9 and 45 ± 20 particles/m^3^, respectively) [7].

There are several ways in which micro- and nanoparticles can enter organisms. In therapeutic and diagnostic applications, nanoparticles are injected directly into the bloodstream. They may also enter an organism through inhalation. In general, the inhaled particle’s size determines its deposition location, i.e., how deep a particle can enter into the airway. Particles with aerodynamic diameters above 100 µm deposit in the upper airways, whereas particles in the 10 to 100 µm range deposit in the oropharynx [8]. Particles with diameters between 5 and 10 µm deposit in the central airway’s mucus layer, whereupon the ciliary movement of the endothelium removes the particles from the airway [8]. Only particles below approximately 5 µm are able to reach the alveoli, whereupon clearance from the lung by macrophage phagocytosis usually takes place [8,9]. Particles with diameters between approximately 0.2 and 0.5 µm are not deposited to a great degree, while particles in the 5 to 100 nm range mainly deposit in the alveoli, and even smaller nanoparticles deposit in the trachea–bronchial region and the upper airways [9]. Location and deposition rate are strongly related to the mechanisms of particle capture. Large particles are mainly deposited due to the inertial mechanism or direct interception, while small particles are captured due to Brownian motion. None of the mentioned mechanisms are significant for particles within the range of 0.2–0.5 µm, which results in their low deposition rate. Inhaled nanoparticles can even cross the alveolar–capillary barrier and directly affect the cardiovascular system [10].

Exposure via ingestion to microplastics present in various environmental compartments may occur either directly or indirectly via the food chain or drinking water [11]. Direct oral exposure to the nanoplastics suspended in the air may occur when deposited inhaled nanoplastics are removed from the lungs by the mucociliary escalator, end up in the oropharynx, and are eventually ingested.

The ways described above in which micro- and nanoparticles enter the human body show that they have contact with various body fluids (saliva, nasal and bronchial mucus, lymph fluid, and blood). The main component of all the aforementioned fluids is water. They also contain various amounts of proteins (enzymes, antibodies, mucins, etc.), lipids, and minerals (sodium, potassium, chloride, and bicarbonate), which are related to their functions and lead to differences in their rheological properties.

The inhalation of micro- and nanoplastics may lead to a number of health implications, ranging from irritation to the onset of cancer in cases of chronic exposure [12]. Other consequences may include immediate asthma-like reactions. Longer exposure may cause inflammatory reactions and fibrotic changes on different levels of the bronchial tree, including chronic bronchitis, lung disorders such as extrinsic allergic alveolitis and chronic pneumonia, pulmonary emphysema, and the development of interstitial lung diseases (coughing, difficulty breathing, and reduction in lung capacity). Moreover, the presence of microplastics in the lungs may lead to oxidative stress and the formation of reactive oxygen species with the potential to damage cells (cytotoxic effects) and cause autoimmune diseases [13].

The ingestion of microplastics increases the risk of diabetes [14] and heart diseases, [15] and weakens immunity [16].

The most essential functions of body fluids are well known, but the effects of their rheological properties on these functions are only sometimes sufficiently recognized. An appropriate “viscosity” of body fluids is necessary in order to create thin protective layers on the surfaces of the mouth, eyes, nose, and respiratory tract against the ingress of pathogens. The effects of microplastics on the rheological properties of saliva and mucus have not been sufficiently recognised so far. However, several papers investigating the impact of nanoparticles on the rheological properties of body fluids have recently been published.

In this study, both saliva and mucus models were used to investigate the effects of the presence of two microplastics, namely, PE (polyethylene) and PS (polystyrene), on their rheology. The reason for choosing these particular microplastics was their significant share in global production. In 2021, the worldwide production of plastic materials reached 390.7 million metric tonnes, of which almost 30% was PE and 3.5% PS [17]. On this basis, the potential impact of microplastics on the functions of saliva and mucus was estimated. According to the authors, no study of this kind has been conducted previously.

## 2. Materials and Methods

### 2.1. Saliva

The properties of saliva (composition, rheology, and flow) are strongly affected by factors like the conditions of their collection, handling, and preservation; age; gender; health status; and even the emotional stress of the saliva donor [18] Therefore, we decided to use artificial saliva in our study.

Artificial saliva was obtained based on the model described by Christersson et al. [19]. The following components—benzalkonium chlorides (12060-5G, Sigma Aldrich, Poznań, Poland) (0.02 g/L), EDTA (E9884-100G, Sigma Aldrich, Poznań, Poland) (0.5 g/L), NaF (1179870000, Chempur, Piekary Śląskie, Poland) (0.0042 g/L), xsylitol (X3375-25G, Sigma Aldrich, Poznań, Poland) (20 g/L), methylparaben (H5501-100G, Sigma Aldrich, Poznań, Poland) (1 g/L), and mucins (type II) (M2378-500G, Sigma-Aldrich, Poznań, Poland) (35 g/L)—were dissolved in deionised water and then placed on a magnetic stirrer (500 rpm) for 2 h. The pH of the solution (7.00) was determined using NaOH or HCl. The sample was stored and sealed at 4 °C.

### 2.2. Mucus

Similarly, we decided to use artificial mucus in our research to obtain reproducible results. From the number of mucus models tested in our previous studies [20,21,22], we chose model [13] for further research. The mucins type II (M2378-500G, Sigma Aldrich, Poznań, Poland) (200 g/L) and NaN_3_ (792770110, POCH, Gliwice, Poland) (0.01 g/L) were dissolved in deionised water and then placed on a magnetic stirrer (500 rpm) for 2 h. Afterward, the pH of the solution (7.4) was adjusted with NaOH or HCl. The mucus was stored in a closed chamber at 4 °C.

### 2.3. Microplastics

The microplastic particles used in the experiments were manufactured by Cosphereic LLC (Goleta, CA, USA). Three types of PE microspheres with diameters of 0.74–4.99 μm, 38–45 μm, and 96–106 μm were used. The particles were in the form of dry powder and were hydrophobic. The density of the particles was 0.96 g/cm^3^.

In our study, we also used two types of PS particles with diameters in the ranges of 9.5–11.5 μm and 38–48 μm. The density of the particles was 1.07 g/cm^3^.

### 2.4. Methodology

The influence of microplastics on the rheological properties of artificial saliva and mucus was determined. Both models of body fluids were prepared the day before the examination. Before adding the microplastic particles, the model body fluids were brought to room temperature by heating in a water bath. PS and PE microparticles were added to the body fluids to achieve a concentration of 6 particles/mL [23]. This concentration of microplastics is found in bottled water. We therefore assumed that we could expect a similar concentration of microplastics in the saliva after exposure to bottled water. This concentration was almost 10 times lower than the concentration of microplastics found in human sputum [24]. However, since microplastics in mucus can accumulate over the time the mucus remains in the respiratory tract, and its occurrence strongly depends on the quality of the air we breathe, we adopted the concentration of microplastics in bottled water as a model concentration to which we are exposed. We also assumed that, if we were to observe an effect at a lower concentration of microplastics, it would be very likely that a similar effect would also occur at a higher concentration of microplastics, which can be found in sputum. The appropriate concentrations of PE and PS particles in the model of saliva and mucus were obtained by assuming (based on the datasheet of PE and PS particles) the number of particles in 1 g of the product (Table 1). A homogeneous microplastic suspension was assumed to be obtained after 15 min of mixing the model body fluids with microplastic particles. The rheological properties (flow curve and the dependence of viscosity as a function of shear stress) were examined with an oscillation rheometer (MCR102, Anton Paar, Graz, Austria) equipped with a Peltier system in a plate–plate system for a gap 1 mm wide. The tests were carried out at 36.6 °C and 40 °C, corresponding to healthy and sick humans. The second temperature was chosen to be high enough for a noticeable difference in viscosity to be observed, and to be distinctly lower than the temperature of protein denaturation at the same time. The rheometer was operated using RheoCompass 1.25 software from Anton Paar. The oscillating rheometer enabled the determination of flow curves (the relationship between shear stress and shear rate) by shearing a liquid sample in the gap between measuring elements rotating relative to each other (e.g., two plates or a cone and a plate). Changing the rotational frequency of the measuring element forced a specific shear rate, leading to shear stresses in the analyzed sample. Shear stresses were determined from the torque acting on the axis of the measuring element.

The appropriate volumes of model saliva and mucus, both pure and with microplastics, were applied using an automatic pipette to the bottom plate of the rheometer. Prior to the rheological measurement, all analyzed samples were subjected to the same preparation procedure, considering the mixing time (1 h), stirrer speed (100 rpm), and temperature (22 °C—room temperature). In addition, to ensure the exact ordering of mucin chains in all samples, the rheometer operation included two intervals following one another at the time of rheological measurement. In the first interval, the sample was exposed to a constant shear rate for 2 min (to organise the mucin chains), while in the second, the actual measurement was carried out. The shear rate range during the second interval was 1–200 s^−1^, which corresponds to typical activities involving saliva (4 s^−1^ corresponds to the movement of particles across the tongue; 60 s^−1^ corresponds to swallowing; and 160 s^−1^ to speech, whilst shear rates of 10–500 s^−1^ have been proposed to reflect the shear during eating [25]). This range is also typical for processes occurring in human respiratory mucus, except for sneezing and coughing [26].

Like natural saliva and mucus, the saliva and mucus models contained mucins. Mucin chains have lengths of several micrometres, diameters of 3–10 nm, and masses in the range of 10–40 MDa. The degree of mixing is essential in rheological measurements for compounds with chain structures. Depending on the degree of mixing, long chains can be more or less tangled and more or less arranged, directly translating into apparent viscosity. Less entanglement and better alignment of the chains lower the apparent viscosity. The sample preparation procedure used by us, as well as the two-interval measurement procedure, eliminated the potential impact of the unequal mixing of the analyzed samples on the rheological measurement result. Each measurement was repeated at least three times, and the presented results are the arithmetic mean of the obtained results.

The least squares method was used to obtain constants in the Ostwald–de Waele Power law rheological model:

(1) $$\tau =k{\dot{\gamma }}^{n}$$

(2) $$\mu =k{\dot{\gamma }}^{n-1}$$

where *τ* is the shear stress, *μ* is the apparent viscosity, *k* is the flow consistency index, and *n* is the flow behavior index. Equation (1) describes the flow curve, while Equation (2) is derived from Equation (1) by comparison with the relation defining apparent viscosity:

(3) $$\tau =\mu \dot{\gamma }$$

The flow behavior index, *n*, is dimensionless, while the dimensions of the flow consistency index, *k*, depend on *n*. For pseudoplastic (shear thinning) fluids, *n* < 1. When *n* > 1, the fluid is dilatant rather than pseudoplastic. If *n* = 1, the fluid is Newtonian. In this case, *k* = *μ*.

An analysis was carried out using the Matlab R2022b Curve Fitting Toolbox. Nonlinear least-squares were the most appropriate for estimating model coefficients in our case.

A nonlinear model has the following matrix form:

*y* = *f*(*X*,*β*) + *ε* (4)

where *f* is a given nonlinear function, *y* is an *n*-by-1 vector of response data, *β* is an *m*-by-1 vector of coefficients, *X* is an *n*-by-*m* matrix of explanatory variables, and *ε* denotes an *n*-by-1 vector of unknown errors of specification.

The Curve Fitting Toolbox used the following iterative approach to calculate the coefficients:

1. Choice of the initial coefficient values;
2. Calculation of the fitted curve for the current set of coefficients. The fitted response value *ŷ* is given by *ŷ* = *f*(*X*,*β*) and is calculated using the Jacobian of *f*(*X*,*β*). The Jacobian of *f*(*X*,*β*) is defined as a matrix of partial derivatives taken concerning the coefficients in *β*;
3. Updating the values of the coefficients using a Trust-region algorithm [27].

The regression was performed for Equation (1). However, the regression for Equation (2) provided exactly the same values for the flow behavior and flow consistency indices. However, the calculated values of the correlation coefficient were different.

We have performed regression for several more advanced rheological models of pseudoplastic fluids, but have not found better agreement with the experimental data. The tested models are summarized in Table 2.

## 3. Results and Discussion

### 3.1. Rheological Properties of Saliva

**Figure 1:**
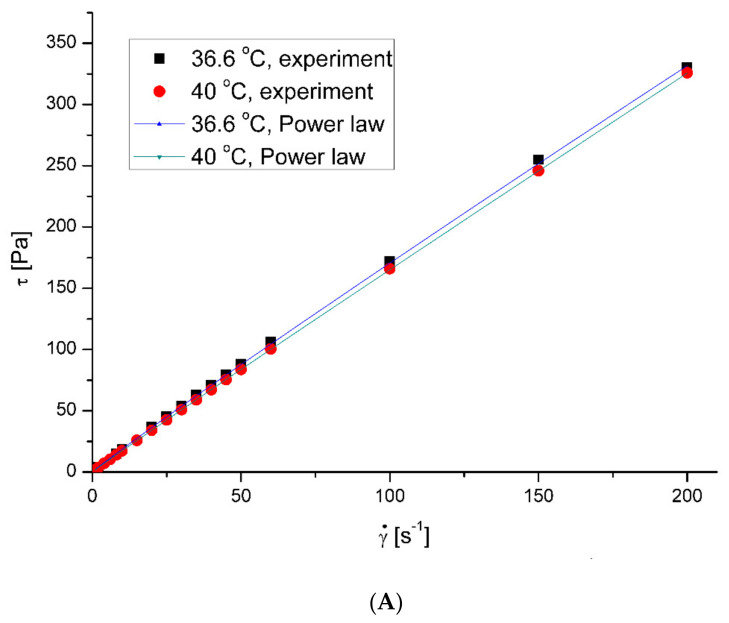

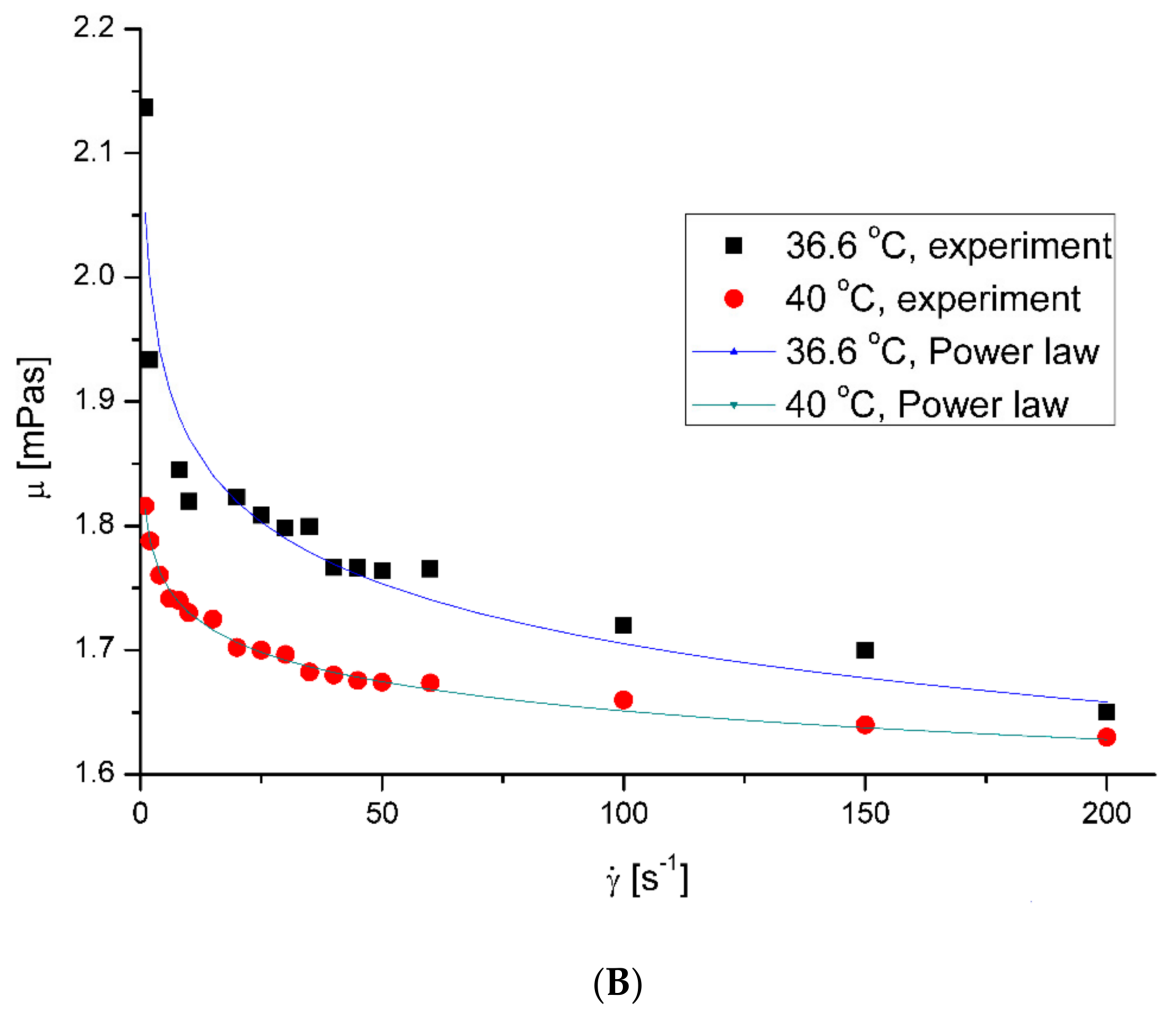
Flow curves (**A**) and apparent viscosity (**B**) for model saliva. Shear stress (Pa) and apparent viscosity (mPas) were measured as a function of shear rate (s^−1^) at 36.6 and 40 °C in an oscillation rheometer.

**Figure 2:**
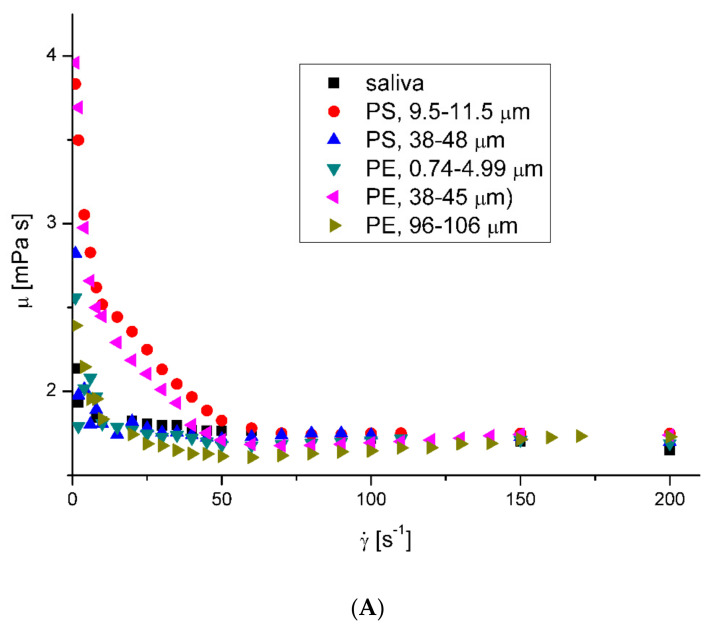

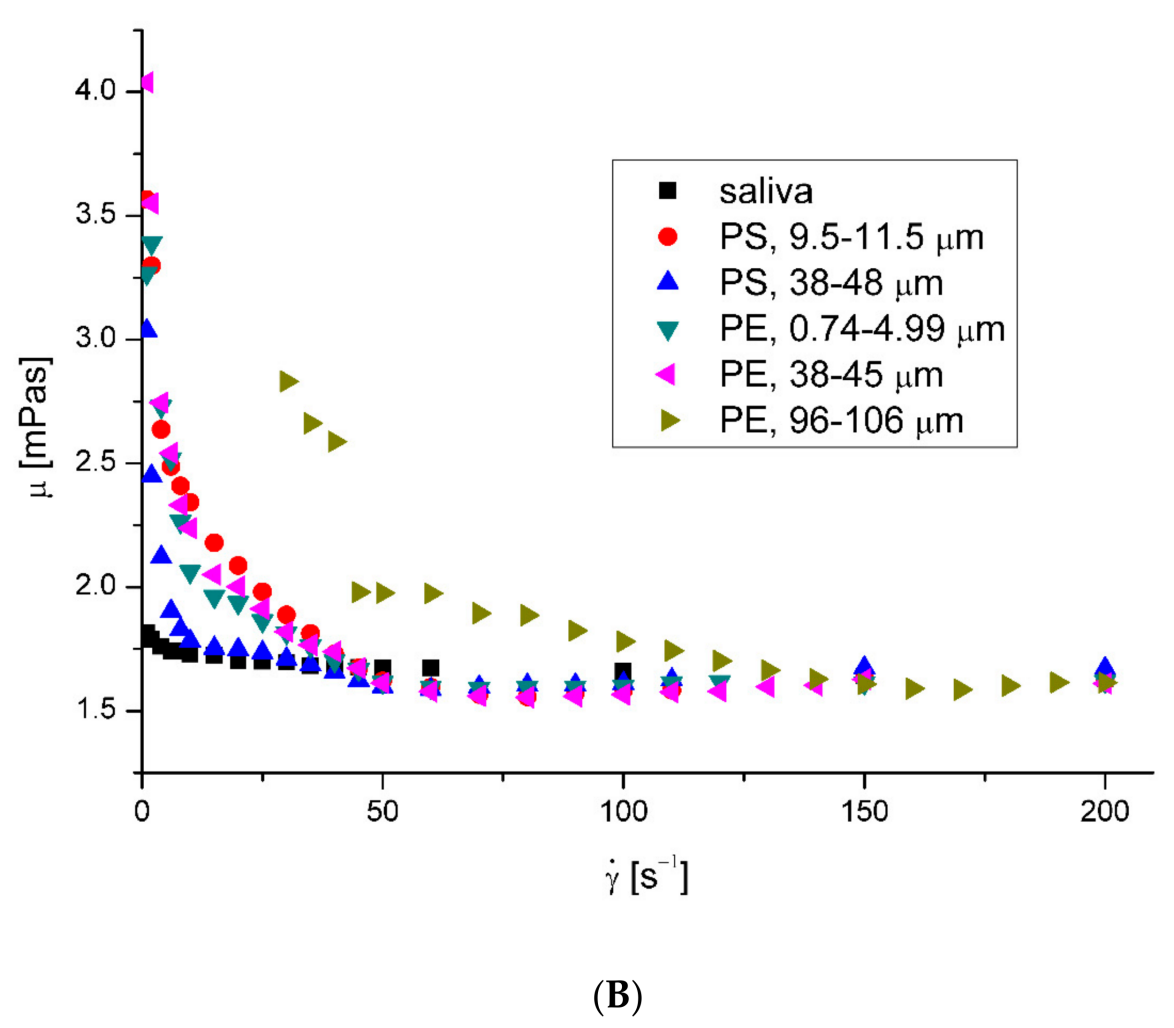
Effects of plastic microparticles on the apparent viscosity of model saliva at 36.6 °C (**A**) and 40 °C (**B**). The apparent viscosity (mPas) was measured as a function of the shear rate (s^−1^) in the oscillation rheometer.

The saliva model behaved slightly like a pseudoplastic fluid. The viscosity decreased with the increase in the shear rate at all examined temperatures. However, the flow curves—shear stress *τ* vs. shear rate $\dot{\gamma }$ (Figure 1A)—were almost straight lines, which is characteristic of Newtonian fluids. Figure 1B presents the effect of the shear rate on the saliva’s apparent viscosity, *μ*. Figure 2 shows the microplastics’ effect on the saliva’s apparent viscosity at 36.6 °C and 40 °C, respectively. The presence of microplastics in artificial saliva increased the viscosity for low shear rates. The effect was visibly weaker with an increasing shear rate, which is typical behavior for solid particle suspensions in fluids [28]. The least squares method regression to the Ostwald–de Waele Power law mode showed that the estimated flow behavior index of pure saliva, *n*, equalled 0.9598 and 0.9797 for temperatures of 36.6 °C and 40 °C, respectively.

The increase in fluid velocity and the presence of microparticles also caused non-Newtonian behavior in the saliva, dropping the value of the flow index noticeably (Table 3).

Studies have shown that periodontal disease and the appearance of caries depend on the saliva’s viscosity. Biesbrock et al. [29] and Yas and Radhi [30] showed that the degree of caries increases with an increase in salivary viscosity. The research did not indicate the cause or the effect. Does high saliva viscosity induce caries, or do advanced caries increase saliva viscosity? Nevertheless, this allows us to conclude that the change in salivary rheology caused by microplastics will not remain inconsequential for human health, although the possible effects may be difficult to predict without ethically questionable clinical trials.

Moreover, while the viscosity of saliva increases, the bacterial co-aggregation decreases, leading to disruption in oral clearance and, consequently, even increases in the likelihood of aspiration pneumonia and cardiovascular diseases, especially in the elderly [31].

### 3.2. Rheological Properties of Mucus

**Figure 3:**
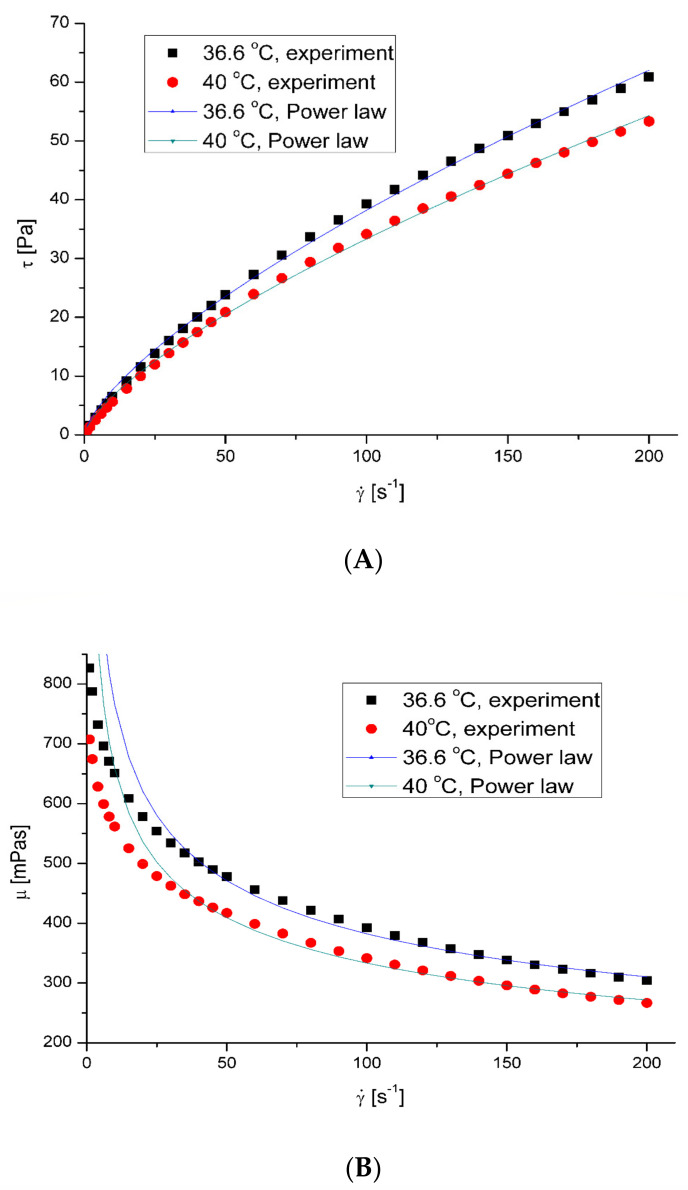
Flow curves (**A**) and apparent viscosity (**B**) for the model mucus. Shear stress (Pa) and apparent viscosity (mPas) were measured as a function of shear rate (s^−1^) at 36.6 and 40 °C using an oscillation rheometer.

The mucus model behaved like a pseudoplastic fluid without yield stress (Figure 3). The non-Newtonian character was observed for clear mucus. The addition of the microplastic particles did not influence the value of the flow index (Table 4). However, in all cases, adding microplastics to mucus led to decreased apparent viscosity.

**Figure 4:**
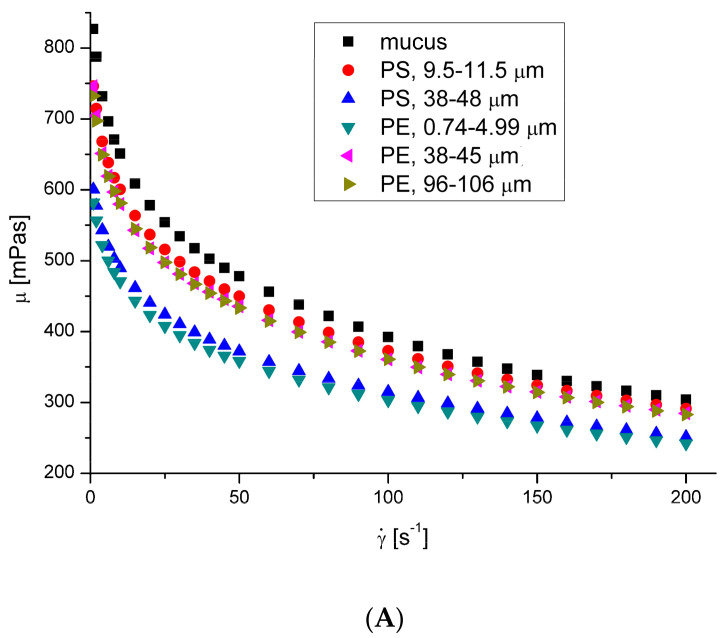

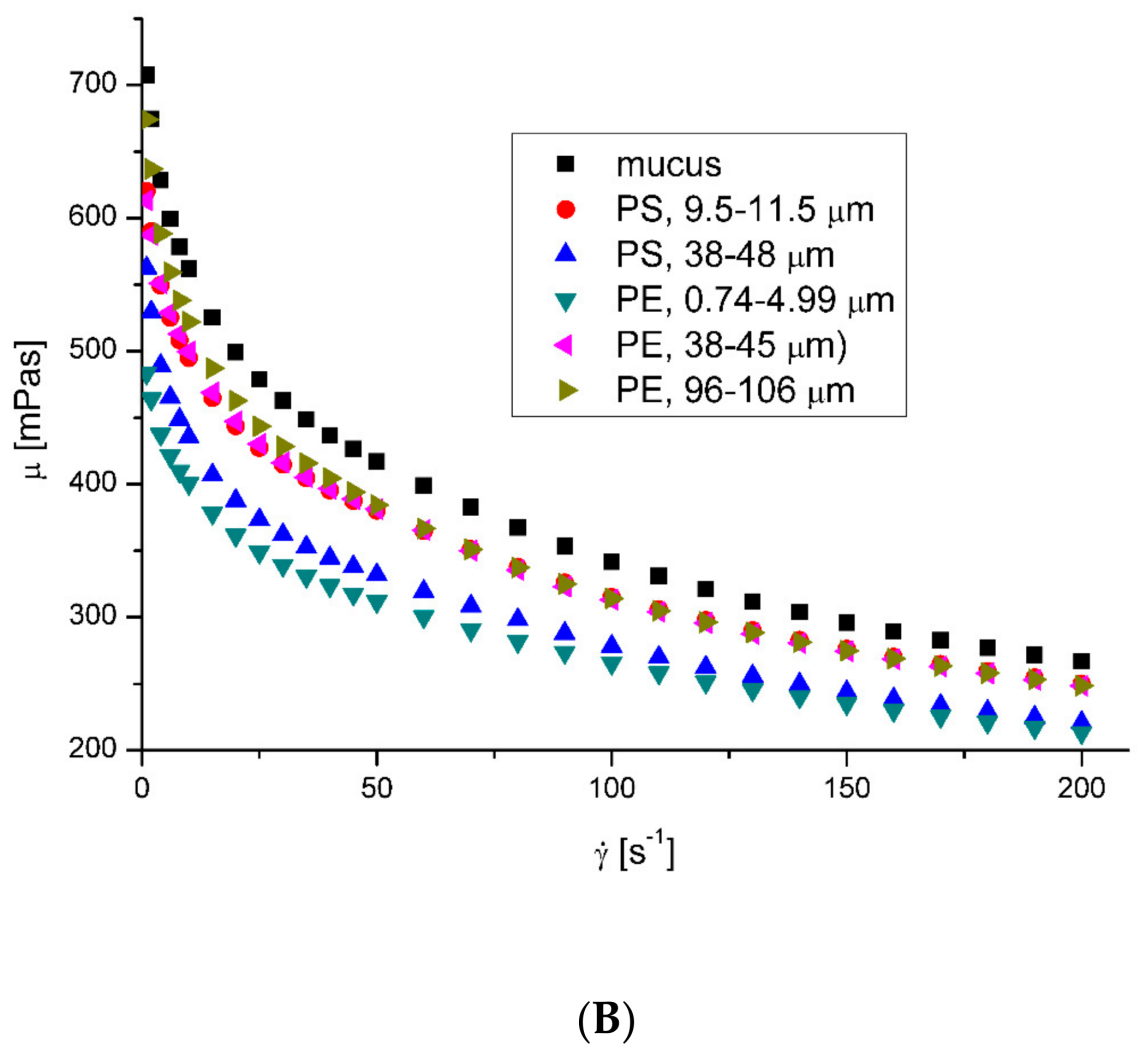
Effect of plastic microparticles on the apparent viscosity of model saliva at 36.6 °C (**A**) and 40 °C (**B**). The apparent viscosity (mPas) was measured as a function of shear rate (s^−1^) in the oscillation rheometer.

The effects of nanoparticles on the apparent viscosity of artificial mucus are presented in Figure 4. A decrease in fluid viscosity by adding suspended particles is not typical behavior, but it has been observed in rare cases for complex fluids [32,33,34]. Ben-David et al. [35] recently showed that in jellyfish mucus, the microsized plastic particles do not form a suspension, but are captured by the mucin network. This is a potential reason for changing its mechanical properties, which may affect its mechanical properties and result in a decrease in the apparent viscosity of mucus. Moreover, there is no explicit relation between the decreasing effect of the presence of microplastics in mucus and their size, suggesting a complex interaction mechanism. A decreasing effect of microparticles on mucus viscosity was observed for the whole range of investigated shear rates. A decrease in mucus viscosity generally negatively affects its protective function. A reduction in viscosity results in an increase in the diffusion coefficient of particles and pathogens possibly present in mucus, and thus increases the possibility of reaching the surface of the epithelium and further being transported into the body. The relation between the diffusion coefficient, *D*, and fluid viscosity is *D*~*μ*^a^, where *a* usually varies from −0.5 to −1 [36]. On the other hand, lower viscosity facilitates the removal of mucus from the respiratory tract by coughing or sneezing.

## 4. Conclusions

The investigated artificial body fluids are a valuable tool for estimating the threat arising from exposure to airborne particles, pointing to a direction for future research. In comparison to natural biological fluids, they have distinctly fewer ingredients, but they always have the same composition, which allows us to obtain universal results. The results show that non-Newtonian, shear-thinning body fluids, i.e., mucus or saliva, react to the presence of plastic particles, changing their apparent viscosity. To confirm the hypothesis that particle–mucin interactions cause the non-trivial behavior of microplastic suspensions in mucus, we intend to perform molecular dynamics calculations. The effect caused by the presence of particles depends on the interaction of the particle with the mucin and the concentration of mucins. Changing the rheological properties of body fluids due to the presence of exogenous particles may cause severe disorders in the body. Changing the viscosity of fluids leads to changes in fluid mechanics, which, in the case of body fluids whose functions are closely related to movement, can have disastrous consequences. However, determining the consequences of changes in the viscosity of body fluids for the body’s function requires research into medicine or biology, which goes far beyond engineering research. Nevertheless, research on model body fluids may give rise to a public discussion on the potential dangers of ubiquitous exposure to microparticles, not just plastic. They are also a valuable tool for estimating threats and a way to conduct relatively easy preliminary research before moving on to research using, for example, animals. We will also investigate the influence of micro- and nanoplastics and other airborne particles, such as diesel exhaust particles (DEP) or natural dust, on the rheological properties of other body fluids, particularly blood. Recent work [32] has suggested that interactions between micro- and nanoparticles and erythrocytes are crucial for blood’s rheological properties. Fluids with low mucin concentrations, like saliva or tears [37], behave like typical suspensions, while the behaviors of fluids with high protein concentrations are much more complex.

## Figures and Tables

**Table 1:** Number of particles in 1 g.

Particles	Number of Particles in 1 g
PE 0.74–4.99 μm	7.762 × 10^8^
PE 38–45 μm	2.672 × 10^7^
PE 96–106 μm	2.029 × 10^6^
PS 9.5–11.5 μm	4.182 × 10^8^
PE 38–48 μm	2.378 × 10^7^

**Table 2:** Rheological models.

Model	Formula	Parameters
Prandtl	$\tau =A\;arc\;sin\;sin\;h\;\left(\frac{\dot{\gamma }}{C}\right)\;$	*A* (Pa), *C* (s^−1^)
Powell–Eyring	$\tau =C\dot{\dot{\gamma }+B}\;arc\;sin\;sin\;h\;\left(\frac{\dot{\gamma }}{A}\right)\;$	*A* (s^−1^), B (Pa), *C* (Pas)
Williamson	$\tau =\left(\frac{A}{B+\dot{\gamma }}+\mu_{\infty }\right)\dot{\gamma }$	*A* (Pa), *B* (s^−1^), *m*∞ (Pas)
Sisko	$\tau =A\dot{\gamma }+B{\dot{\gamma }}^{n}$	*A* (Pas), *B* (Pas^n^), *n* (-)

**Table 3:** Constants in the Ostwald–de Waele Power law model and coefficient of determination of the least-squares method for saliva.

Case	*k* (mPas^n^)	*n* (-)	*R* ^2^
Saliva, 36.6 °C	2.052	0.9598	0.8647
Saliva, 40 °C	1.813	0.9797	0.9911
PS, 9.5–11.5 μm, 36.6 °C	3.904	0.8169	0.9918
PS, 9.5–11.5 μm, 40 °C	3.619	0.8041	0.9889
PS, 38–48 μm, 36.6 °C	2.645	0.8782	0.8816
PS, 38–48 μm, 40 °C	2.759	0.8482	0.8891
PE, 0.74–4.99 μm, 36.6 °C	2.514	0.8924	0.9681
PE, 0.74–4.99 μm, 40 °C	3.323	0.8198	0.9809
PE, 38–45 μm, 36.6 °C	4.038	0.7874	0.9876
PE, 38–45 μm, 40 °C	3.980	0.7685	0.9863
PE, 96–106 μm, 36.6 °C	2.407	0.8954	0.9835
PE, 96–106 μm, 40 °C	2.911	0.8551	0.8708

**Table 4:** Constants in the Ostwald–de Waele Power law model and coefficient of determination of least-squares method for mucus.

Case	*k* (mPas^n^)	*n* (-)	*R* ^2^
Mucus, 36.6 °C	1531	0.6987	0.9983
Mucus, 40 °C	1301	0.7043	0.9984
PS, 9.5–11.5 μm, 36.6 °C	1367	0.7122	0.9984
PS, 9.5–11.5 μm,40 °C	1097	0.7244	0.9985
PS, 38–48 μm, 36.6 °C	1032	0.7372	0.9989
PS, 38–48 μm, 40 °C	944	0.7294	0.9987
PE, 0.74–4.99 μm, 36.6 °C	992	0.7374	0.9985
PE, 0.74–4.99 μm, 40 °C	831	0.7470	0.9988
PE, 38–45 μm, 36.6 °C	1305	0.7874	0.9876
PE, 38–45 μm, 40 °C	1173	0.7102	0.9988
PE, 96–106 μm, 36.6 °C	944	0.7484	0.9988
PE, 96–106 μm, 40 °C	1113	0.7205	0.9986

## Data Availability

The data presented in this study are openly available in Zenodo at https://doi.org/10.5281/zenodo.10090802.
